# A critical evaluation of the exotic bird collection of the Šariš Museum in Bardejov, Slovakia

**DOI:** 10.3897/zookeys.776.24462

**Published:** 2018-07-26

**Authors:** Peter Mikula, Alexander Csanády, Martin Hromada

**Affiliations:** 1 Department of Zoology, Faculty of Science, Charles University, Viničná 7, 128 43 Praha 2, Czech Republic Charles University Praha Czech Republic; 2 Department of Biology, Faculty of Humanities and Natural Sciences, University of Presov, 17. novembra 1, 080 01 Prešov, Slovakia University of Prešov Prešov Slovakia; 3 Laboratory and Museum of Evolutionary Ecology, Department of Ecology, Faculty of Humanities and Natural Sciences, University of Presov, 17. novembra 15, 080 01 Prešov, Slovakia University of Zielona Gora Zielona Gora Poland; 4 Faculty of Biological Sciences, University of Zielona Góra, Prof. Z. Szafrana 1, 65–516 Zielona Góra, Poland Charles University Praha Czech Republic

**Keywords:** Aves, biodiversity, museum, ornithological collections, species occurrence data

## Abstract

A collection of exotic birds deposited in the Šariš Museum in Bardejov (SMB), Slovakia, has not been evaluated critically since their deposition. We assessed the accuracy of identification of 465 bird specimens deposited in SMB with native distributions outside of Slovakia. Specimens belonged to 322 species of 82 families and 26 orders. Of the specimen represented, 34 belonged to species considered as ‘near-threatened’ (7.3%), 16 as ‘vulnerable’ (3.4%) and one as ‘endangered’ (0.2%). The SMB collection holds 10 of 28 extant Cuban endemic species and another 11 species endemic to the Caribbean archipelago. Even among birds that are relatively easy to identify, many specimens were identified incorrectly or species identification was missing. Of 465 specimens evaluated, 95 (20.4%) were identified incorrectly or were missing species identification, and another 79 (17%) were identified correctly, but their names have changed over time due to taxonomic shift, thus they required correction.

## Introduction

Natural history collections have long served as a primary data source for addressing fundamental questions in systematics, biogeography, and conservation of organisms. Specimens in such collections represent an important source of documentation of present and past occurrences of species with each specimen being unique and irreplaceable (Winker et al. 1991, Wandeler et al. 2007, Ariño 2010, Kress 2014). Specimens provide a window into evolutionary processes in natural populations, enabling researchers to study evolution on timescales similar to those from long-term field studies or experiments in laboratories (Holmes et al. 2016). However, many natural history collections around the world are at risk in view of declining funding and expenses of adequate upkeep (Winker et al. 1991, 2010, Joseph 2011, Gardner et al. 2014, Krell and Wheeler 2014, Kemp 2015). Many collections, including the most renowned ones, still hold numerous specimens lacking identification or awaiting revision (Winker et al. 1991), to the extent that several bird species new to science are discovered each year (del Hoyo et al. 2013), often through the re-evaluation of museum specimens, mostly using new approaches such as genetic methods (Winker et al. 1991).

Natural ecosystems are currently changing at unprecedented rates owing to human activity, affecting both terrestrial and marine ecosystems (Vitousek et al. 1997, Ellis and Ramankutty 2008, Barnosky et al. 2011, Dirzo et al. 2014, Ceballos et al. 2015). For instance, roughly half of the world’s terrestrial surface area has undergone conversion to grazed land or cultivated crops (Kareiva et al. 2007) and ~60% of the world’s largest terrestrial herbivores are threatened with extinction owing to over-hunting, land-use change, and competition with livestock (Ripple et al. 2015). Although birds tend to be less threatened than other vertebrates (Hoffmann et al. 2010), several bird species have shown recent shifts in distribution and abundance as a result of human-induced environmental change (Thomas et al. 2004, Inger et al. 2015). Most vulnerable are species with small geographic distributions, particularly island species (e.g. Steadman 1995, Blackburn et al. 2004, Hoeck et al. 2011). However, not only rare species are endangered; for instance, over the last three decades, bird populations in Europe declined by ~20%, with many common species suffering steep declines (Kress 2014, Inger et al. 2015).

Museum collections include specimens collected in various places and times that provide important insights into long-term consequences of natural or anthropogenic environmental changes (Glenn et al. 1999, Godoy et al. 2004, Kuhn et al. 2013, Gardner 2014, Kress 2014, Mason and Unitt 2018). The importance of specimens has actually increased recently as specimen collection has been criticised increasingly, thus, obtaining of new voucher specimens is much complicated, often even stopped (Minteer et al. 2014, Waeber et al. 2017). Most museum-based studies are carried out in large, well-known collections as they are more accessible to scientific public (Mearns and Mearns 1998), however, thanks to technological development and public access to the internet, information from all natural history collections, including those smaller and / or less important ones, is becoming much more accessible (e.g. Navarro-Sigüenza et al. 2003, Graham et al. 2004, Peterson et al. 2016). This visibility increases the global importance of local museums and their collections, which were often unavailable to the wider scientific community (Monteiro et al. 2016, 2017).

One of the important European ornithological collections is held by the Natural History Department of the Šaris Museum in Bardejov (SMB), Slovakia (Roselaar 2003, Hromada et al. 2015). This collection was established by the prominent Slovak zoologist, Tibor Weisz, in 1956, with the majority of the bird specimens collected by the department’s founder himself during 1956–1983 (Hromada et al. 2015). The collection is unique in several respects: besides building the general collection, the collector focused on several particular species, obtaining not only the largest series of some species in the world, but also systematically covering with his vouchers the long time period of four to five decades (Hromada et al. 2015). Compared to other collections (Mearns and Mearns 1998), data associated with bird collection in SMB are quite rich, including more measurements and notes, such as data on condition (general health), size of gonads, notes on colouration, and habitat and behaviour; specimens also include other associated voucher material (sternum, stomach, ecto- and endoparasites, egg clutches, etc.) (Tryjanowski et al. 2001, Hromada et al. 2003, Hromada and Klimovičová 2015, Hromada et al. 2015). The ornithological collection is focused predominantly on Slovak birds (5047 specimens of 251 species and 61 families; Hromada et al. 2015), but Weisz collected and maintained also collection of “exotic” species (not occurring in Slovakia). The majority of exotic specimens was collected by Weisz himself and taxidermist Vilém Borůvka (Hromada 2015) during a visit to Cuba (1968); others were received in exchange from foreign collectors (e.g., A. Kovács, Argentina; N. H. Gustafsson, Denmark). The bird collection was first catalogued by Weisz during his work at SMB; species were identified by his peer ornithologist Aristid Mošanský (Hromada 2015), but since that time no update or revision has been carried out.

Hence, our aim is a systematic and critical re-evaluation of the species identities of the exotic bird specimens in SMB collection, for several reasons. (1) In Weisz's time in SMB, it was problematic for Eastern bloc scientists to get good (if any) handbooks on birds, particularly for New World birds. (2) Recently, many bird clades have seen taxonomic revision, often resulting in splits of species and updates in nomenclature. Lack of recent and ongoing comprehensive taxonomic re-evaluation of specimens causes many specimens to have inaccurate names. (3) Despite the collection is held in small, local museum, it contains specimens and/or species important from global point of view, thus, it is crucial to make it publicly available. Therefore, we present here the results of a first detailed review of the exotic birds deposited in SMB, complementing a recent study dedicated to Slovak birds in SMB (Hromada et al. 2015).

## Methods

Because the bulk of the exotic specimens were in exhibition, it was often not possible to base identification work on the in-hand specimens *per se*. Hence, we photographed all exotic bird specimens (i.e. those with distributions outside Slovakia) and data cards associated with data on identification, locality, date, sex, and catalogue number. We then visually inspected all photos and identified species using the online edition of “Handbook of the Birds of the World” (del Hoyo et al. 2017). If species identification was at all questionable, we asked experts (see acknowledgements) and members of online birding communities (e.g. http://www.birdforum.net) for help. All specimens with detailed locality given were georeferenced (see Suppl. material 1).

We were unable to find occurrence records for several specimens. To make information on these specimens as complete as possible, we added locality country and date for some based on our knowledge of when and where Weisz collected specimens. (1) Specimens marked as collected by T. Weisz (i.e. not received in exchange from other collectors) without given locality or date were associated with Cuba and the year 1968 (when Weisz and Borůvka visited Cuba) if their distributions included Cuba. We know of no other visit of Weisz to the Neotropical region nor any specimen exchanges with Cuban origins. (2) Specimens of species endemic to other countries were associated with that country, except for domesticated species (e.g., *Syrmaticusreevesii* (Gray, 1829)). (3) If date of collection was unknown but we had information on date of acquisition in SMB we used that date as in most cases the two dates were the same. The only exception was by 1977 when Weisz registered many old specimens; specimens registered in 1977 were thus left without a date if no date of collection was provided. However, if information on locality and / or date was not found in original documentation but added by us by aforementioned procedure, this was always clearly highlighted for each specimen in Suppl. material 1. Following identification, each species was then checked as to conservation status (IUCN Red List of Threatened Species, version 3.1; http://www.iucnredlist.org/static/categories_criteria_3_1) in the following categories: (1) Least Concern (LC), (2) Near Threatened (NT), (3) Vulnerable (VU), and (4) Endangered (EN).

Finally, we paid special attention to bird specimens originating in Cuba and the Caribbean region, especially those species endemic to this region, according to the online bird checklist, Avibase (http://avibase.bsc-eoc.org/), under “Handbook of the Birds of the World Alive” (del Hoyo et al. 2017) taxonomy. To address the importance of Cuban specimens deposited in SMB, we conducted searches on publicly accessible databases of global vertebrate biodiversity, such as VertNet (http://www.vertnet.org/) and Global Biodiversity Information Facility (GBIF; https://www.gbif.org/; we focused on number of preserved specimens only, excluding, for instance, human observations) (accessed on 25.10.2017).

## Results

**Figure 1.**
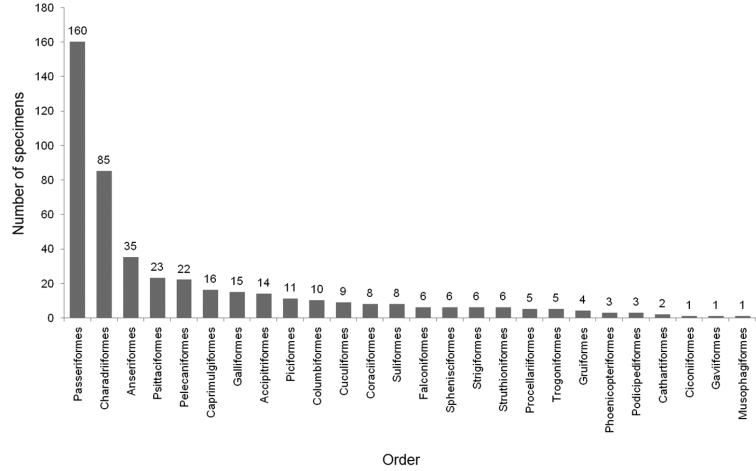
Total number of specimens per order represented in the exotic bird collection of the Šariš Museum in Bardejov, Slovakia.

In total, SMB collection comprises 465 specimens (267 specimens are in public exhibition, 198 in scientific collection) of exotic bird species (for specimen details see Suppl. material 1). We were able to identify 454 specimens to species, in 11 specimens species identification remains questionable. Specimens identified belonged to 322 species, 82 families, and 26 orders. Most families were in the order Passeriformes (40.2%), followed by Charadriiformes (13.4%) and Pelecaniformes (4.9%). The most specimen-rich orders were Passeriformes (34.4%), Charadriiformes (18.3%), and Anseriformes (7.5%). Six orders (Cathartiformes, Ciconiiformes, Gaviiformes, Musophagiformes, Phoenicopteriformes and Podicipediformes) were represented by three or fewer specimens (Figure 1).

SMB holds a collection focused on the birds of Cuba and Caribbean archipelago. This collection holds 21 specimens that pertain to 10 of 28 extant Cuban endemic species and another 21 specimens corresponding to 11 species endemic to the Caribbean archipelago (Table 1).

**Figure 2.**
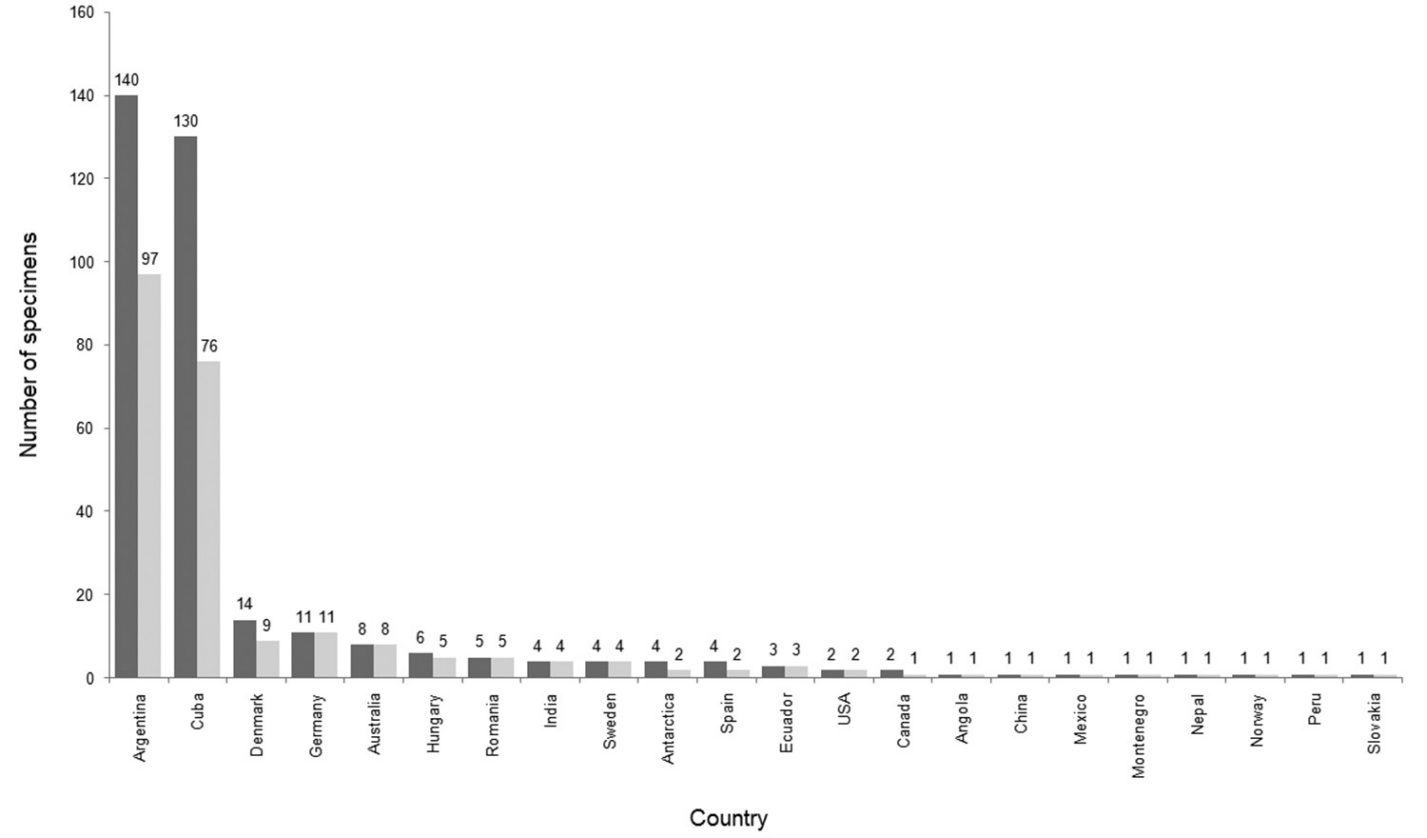
The geographic distribution of the Šariš Museum in Bardejov exotic bird collection with number of bird specimens (dark grey) and species (light grey) collected from each country.

**Figure 3.**
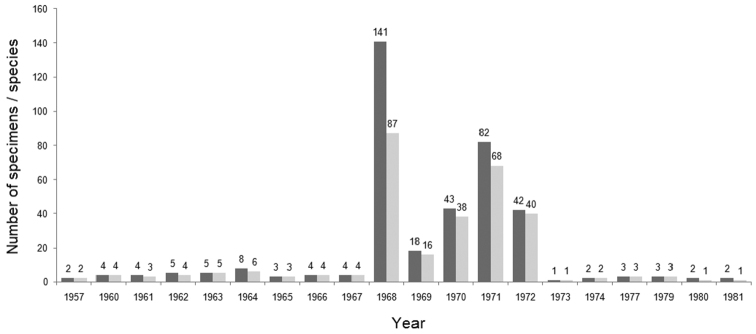
Temporal distribution of numbers of bird specimens (dark grey) and species (light grey) added to the Šariš Museum in Bardejov exotic bird collection during 1957–1981.

Beside Cuba, specimens in the SMB exotic bird collection came from at least 22 countries. Most specimens with identified locality of collection at least on the country level came from Argentina (140), followed by Cuba (130), Denmark (including Greenland) (14), Germany (11), and Australia (8). Two exotic bird species often kept as pets were also included – *Melopsittacusundulatus* (Shaw, 1805) and Serinuscanariaf.domestica (Linnaeus, 1758). We were not able to associate country of collection for 120 specimens (Figure 2). The SMB exotic bird collection covered years 1957–1981. Most specimens were collected in 1968, during the expedition to Cuba and 1970–1972 via exchanges with the Argentine collector, A. Kovács. We were unable to associate year of collection / acquisition year for 86 individuals (Figure 3).

The majority of SMB specimens belonged to species classified by the IUCN Red List as LC (414 specimens, 89%), followed by NT (34 specimens, 7.3%), VU (16 specimens, 3.4%) and EN (one specimen of *Oxyuraleucocephala* (Scopoli, 1769), 0.2%).

Out of 465 specimens, 291 (62.6%) were identified correctly, with scientific names that are still valid; another 69 (14.8%) specimens were correctly identified but names have changed over time. In 10 cases (2.2%), the species was treated as conspecific with other species, but current taxonomy recognised them as separate; in 21 (4.5%) specimens, the identification was incorrect; and 63 (13.6%) specimens had species identification missing at the time of our revision. In 10 cases (2.2%), species identification was previously missing, and we remain unsure of identification; in one case (0.2%), species identification was previously incorrect and we are not sure about the correct identification.

**Table 1.** Bird species endemic to (a) Cuba and (b) the Caribbean region hold by the Šaris Museum in Bardejov, Slovakia, including number of specimens in public exhibition and the scientific collection and their conservation status according to the IUCN Red List. From online biodiversity databases, VertNet and GBIF, we obtained information on numbers each species deposited in other natural history collections worldwide.

Common name	Scientific name	Exhibition	Collection	VertNet^†^	GBIF^†^	IUCN Red List^‡^
**(a) Cuban endemic species**						
Cuban Black Hawk	* Buteogallus gundlachii *	1	0	5 (13)^1^	6 (26)	NT
Cuban Pygmy Owl	* Glaucidium siju *	1	0	205	258	LC
Cuban Trogon	* Priotelus temnurus *	1	1	253	336	LC
Cuban Tody	* Todus multicolor *	1	2	288	354	LC
Cuban Green Woodpecker	* Xiphidiopicus percussus *	1	0	252	340	LC
Cuban Gnatcatcher	* Polioptila lembeyei *	3	0	69	130	LC
Cuban Blackbird	* Ptiloxena atroviolacea *	1	1	7 (12)^2^	15 (169)	LC
Cuban Grassquit	* Phonipara canora *	2	0	0 (48)^3^	14 (346)	LC
Cuban Parakeet	* Psittacara euops *	0	1	7 (73)^4^	11 (128)	VU
Cuban Oriole	* Icterus melanopsis *	5	0	19 (188)^5^	30 (287)	LC
**(b) Carribean endemic species**						
Cuban Emerald	* Chlorostilbon ricordii *	1	2	349	420	LC
Cuban Lizard-cuckoo	* Coccyzus merlini *	1	1	28 (222)^5^	2 (380)	LC
West Indian Woodpecker	* Melanerpes superciliaris *	2	0	472	623	LC
Cuban Amazon	* Amazona leucocephala *	1	2	349	501	NT
Loggerhead Kingbird	* Tyrannus caudifasciatus *	1	1	614	914	LC
La Sagra‘s Flycatcher	* Myiarchus sagrae *	1	0	299 (14)^6^	437 (16)	LC
Cuban Pewee	* Contopus caribaeus *	0	1	351	527	LC
Cuban Crow	* Corvus nasicus *	1	0	81	138	LC
Red-Legged Thrush	* Turdus plumbeus *	1	1	548	1040	LC
Cuban Bullfinch	* Melopyrrha nigra *	3	0	224	325	LC
Greater Antillean Grackle	* Quiscalus niger *	1	0	804	1037	LC

^†^Number in brackets is a number of results from search using alternative species names: ^1^*Buteogallusanthracinus*, ^2^*Divesatroviolaceus*, both only with origin from Cuba; ^3^*Tiariscanorus*; *^4^Aratinga euops*; ^5^*Icterusdominicensis*; ^5^*Saurotheramerlini*; ^6^*Myiarchusstolidus* with origin from Cuba ^‡^LC – Least concern, NT – Near threatened, VU – Vulnerable, EN – Endangered.

## Discussion

### Value of the SMB exotic bird collection

The Caribbean archipelago is known for high levels of species endemism, and forms part of a world hotspot region of endemism (Orme et al. 2005). Cuba is the largest island of the Caribbean region, with 28 out of 180 extant bird species endemic to this island. Many more endemic species vanished during subrecent to recent times owing to human activity (Milberg and Tyrberg 1993). Terrestrial ecosystems of Caribbean region are still under increasing pressure from human populations appropriating large portions of their distributional areas (Haberl et al. 2007). The SMB collection is thus valuable because it harbours one-third of all species endemic to Cuba, some of which are poorly represented in world collections, including one specimen of *Buteogallusgundlachii* (Cabanis 1855) for which only a very small number of specimens is registered on VertNet and GBIF (Table 1). Specimen data on rare species of Caribbean region from other collections were recently used, for instance, for species distribution modelling of charismatic and presumably extinct *Campephilusprincipalis* (Lammertink 1995, Gotelli et al. 2012).

The SMB exotic bird collection included 51 specimens of 33 species classified as near threatened or higher threat categories. We highlight a male specimen of the endangered species *Oxyuraleucocephala*, which populations undergone fragmentation and rapid declines in recent decades, resulting in loss of genetic diversity (Muñoz-Fuentes et al. 2005). The specimen was collected in the breeding season (8. June) 1960 in Soltvadkert, Hungary. The last confirmed breeding of this species in Hungary was in 1961 (Green and Anstey 1992) and the breeding population of the species is now extinct there (Birdlife International 2017). From other examples, the collections included specimens of the vulnerable species *Phoenicoparrusandinus* (Philippi, 1854) and *Buteoventralis* Gould, 1837 (both collected from Argentina), with only seven and 18 specimens, respectively, of these species from Argentina registered in GBIF (accessed on 2.11.2017).

We showed that many specimens in the SMB collection are rare and poorly represented in scientific collections in other museums. Hence, our results may help recognise the importance of the collection by responsible authorities and take actions that would provide adequate maintenance of the specimens in the collection. Despite the fact that many of the important specimens are on public display, we do not expect that this could affect their availability for scientific research because SMB is open to making the collection available to the scientific as well as broader community, e. g. in order to provide data to international databases. However, public display is a danger to specimens in several ways (e. g. damage by pests, fading, etc.). Fortunately, the museum regime currently provides protection to some extent to specimens placed in public exhibition because the direct sunlight in the exhibition is eliminated (no windows are present there) and the artificial light is switched on only when visitors are inside (number of visitors is low in general).

Moreover, specimens from the SMB exotic bird collection enabled description of several new species of obligate bird parasites, particularly quill mites (Acari: Syringophilidae), living within the feather calamus (Skoracki and Sikora 2002, 2004, Hromada and Klimovičová 2015). Probably most importantly, the first record of a parasitic quill mite from a palaeognath bird, *Eudromiaelegans* Geoffroy Saint-Hilaire, 1832, named *Tinamiphilopsiselegans* Skoracki & Sikora, 2004, was described in 2004 as a new genus (Skoracki and Sikora 2004). Until then, quill mites were known only in neognath species (Skoracki and Sikora 2004).

### Identification issues

Correct species identifications are essential in research, with key implications, for instance, for systematics, biogeography, and conservation (Johansson et al. 2013, 2018, Tritsch et al. 2017). Accuracy of identifications varies significantly between taxa, even when identified by experts; in some groups, such as insects, overall accuracy may be quite low (Austen et al. 2016). The situation is even more problematic in plants for which as many as half of plant specimens in tropical collections may have wrong identifications (Goodwin et al. 2015). In contrast, birds are relatively easy to identify (Panhuis et al. 2001, Edwards et al. 2005).

Nonetheless, we found that 95 specimens (20.4%) deposited in the SMB exotic bird collection were identified incorrectly or were missing species identification at the time of our revision. This gap can be attributed to limited access to identification literature at those times, mainly for scientists from the Eastern Bloc. Another 79 specimens (17%) had correct names, but they needed update. Altogether, then, more than one-third of specimens in the SMB exotic bird collection had names that were incorrect according current taxonomy and nomenclature.

One of the best examples that critical evaluation of accuracy of specimen names is important is the specimen of the endemic and near threatened Cuban species *Buteogallusgundlachii*, which was originally identified as Buteo (Geranoaetus) albicaudatus (Vieillot, 1816). Hence, even in collections of animals groups that are relatively easy to identify, accuracy of specimen names may be not as high as expected. Issues (i.e. missing, mistaken and outdated identifications) are expected to accumulate and concentrated in: (1) old collections without continuous evaluation of specimens; (2) local collections focused on local biota, but having some exotic voucher material; and (3) collections managed by amateurs or by experts of unbalanced expertise.

## Supplementary materials

Supplementary material 1 Table S1 Data type: museum records Explanation note: Exotic bird specimens held by the Šaris Museum in Bardejov, Slovakia, including their updated identification, previous identification provided by museum, family and order of bird specimen, conservation status according IUCN, country / region of specimen origin, locality of collection, acquisition number and year, inventory and notebook number, date of collection, and whether specimen is located in public exhibition or scientific collection. ? – information on locality and / or date was not found but we added this information for some specimens based on our knowledge of when and where Weisz collected specimens (for more details see method section). This is an open access article distributed under the terms of the Creative Commons Attribution License (CC BY 4.0), which permits unrestricted use, distribution, and reproduction in any medium, provided the original author and source are credited.Peter Mikula, Alexander Csanády, Martin Hromada
